# Analysis of Non-Structural Carbohydrates and Xylem Anatomy of Leaf Petioles Offers New Insights in the Drought Response of Two Grapevine Cultivars

**DOI:** 10.3390/ijms21041457

**Published:** 2020-02-20

**Authors:** Rachele Falchi, Elisa Petrussa, Enrico Braidot, Paolo Sivilotti, Francesco Boscutti, Marco Vuerich, Carla Calligaro, Antonio Filippi, José Carlos Herrera, Paolo Sabbatini, Marco Zancani, Andrea Nardini, Enrico Peterlunger, Valentino Casolo

**Affiliations:** 1 Department of Agricultural Food, Animal and Environmental Sciences, University of Udine, via delle Scienze 206, 33100 Udine, Italy; rachele.falchi@uniud.it (R.F.); elisa.petrussa@uniud.it (E.P.); enrico.braidot@uniud.it (E.B.); paolo.sivilotti@uniud.it (P.S.); francesco.boscutti@uniud.it (F.B.); marco.vuerich@gmail.com (M.V.); carla.calligaro@uniud.it (C.C.); antonio.filippi@uniud.it (A.F.); marco.zancani@uniud.it (M.Z.); enrico.peterlunger@uniud.it (E.P.); 2 Institute of Viticulture and Pomology, Department of Crop Sciences, University of Natural Resources and Life Sciences Vienna (BOKU), Konrad-Lorenz Straβe 24, 3430 Tulln, Austria; jose.herrera@boku.ac.at; 3 Department of Horticulture, Michigan State University, 1066 Bogue Street, East Lansing, MI 48824, USA; sabbatin@msu.edu; 4 Department of Life Sciences, University of Trieste, via Licio Giorgieri, 5, 34127 Trieste, Italy; nardini@units.it

**Keywords:** Cabernet Sauvignon, Syrah, glucose, maltose, starch, sucrose, conduits area, drought, recovery

## Abstract

In grapevine, the anatomy of xylem conduits and the non-structural carbohydrates (NSCs) content of the associated living parenchyma are expected to influence water transport under water limitation. In fact, both NSC and xylem features play a role in plant recovery from drought stress. We evaluated these traits in petioles of Cabernet Sauvignon (CS) and Syrah (SY) cultivars during water stress (WS) and recovery. In CS, the stress response was associated to NSC consumption, supporting the hypothesis that starch mobilization is related to an increased supply of maltose and sucrose, putatively involved in drought stress responses at the xylem level. In contrast, in SY, the WS-induced increase in the latter soluble NSCs was maintained even 2 days after re-watering, suggesting a different pattern of utilization of NSC resources. Interestingly, the anatomical analysis revealed that conduits are constitutively wider in SY in well-watered (WW) plants, and that water stress led to the production of narrower conduits only in this cultivar.

## 1. Introduction

The health decline of woody plants subjected to drought has been ascribed to two main processes: hydraulic failure and carbon starvation [1]. The first process results in impaired water transport due to xylem embolism formation. In some plants, the occurrence of early embolism in distal sectors leads to the sacrifice of short-lived organs (i.e., leaves), thus reducing transpiring surface and energy needs. This strategy was postulated for the first time by Zimmerman [2], and it is known as “vulnerability segmentation”. This concept was later tested using grapevine as a model plant, because of its marked susceptibility to petiole xylem embolism, and leaf shedding has been suggested to be a strategy to prevent embolism propagation from petioles to stem vascular tissues [3,4].

Plants suffering reduced hydraulic efficiency also undergo a decrease in photosynthesis due to stomatal closure, leading to the consumption of non-structural carbohydrate (NSC) reserves. It has been suggested that the hydraulic behavior of different plant species influences the occurrence of this phenomenon [5,6]. In particular, isohydric species are considered to be more exposed to carbon starvation risk when compared to anisohydric ones, due to the prompt stomata closure in the case of water deficit [1,7]. However, this assumption needs to be taken with caution, considering that the isohydric/anisohydric definition has been recently revised, pointing out that this classification can be significantly affected by the environment and other intrinsic traits of the plant [8,9,10].

In woody plants, xylem water transport can be restored after a water stress period by producing new xylem conduits [11], and/or retrieving water in the xylem to refill embolized conduits [12,13,14,15,16,17]. It has been hypothesized that the latter process involves soluble NSCs decreasing the osmotic potential in the embolized xylem, thus promoting water movement from the nearest parenchymatic cells to the gas-filled conduits [18,19,20]. The degradation of starch seems to play a major role in embolism repair by providing high levels of sucrose in parenchyma cells adjacent to xylem conduits [20,21,22,23]. Furthermore, enhanced concentrations of glucose, fructose, sucrose and other products of starch degradation (e.g., maltose) have been recently observed in the xylem sap of poplars subjected to drought [24].

Grapevine is cultivated worldwide in several contrasting climates. Increasing mean daily temperatures have been recorded over the past decades in many vine-growing regions, with seasonal trends characterized by milder winters and more frequent summer droughts. Due to the economic importance of this crop, such changes have stimulated investigations of grapevine responses to drought [25,26,27,28,29]. Indeed, the global scarcity of water resources is becoming a major limiting factor in grapevine cultivation [30]. Drought stress responses are strictly dependent on the specific adaptation of each cultivar to environmental conditions [31]. Chouzouri et al. [32] reported that xylem anatomical traits significantly influenced gas exchange and vulnerability to embolism. In particular, the number and the size of xylem conduits are key factors in modulating the hydraulic responses in grapevines. Recent studies confirmed that NSCs have a crucial role in sustaining grapevine metabolism under drought conditions [33]. Falchi et al. [34] reported that dormant canes of cv. Syrah (SY) and Cabernet Sauvignon (CS), subjected to an early or late drought stress in the previous growing season changed their soluble NSC pools in both wood and bark. Accordingly, after recovering from water stress, drought affected the leaf petiole transcriptome and, in particular, genes related to sugar metabolism [28].

Among the adaptive strategies adopted by anisohydric grapevine cultivars under drought stress, leaf osmotic adjustment has been proposed to occur by active accumulation of carbohydrates and other osmolytes together with anatomical trait modulation [26]. However, contrasting results have been reported in the literature regarding the contribution of NSC to active osmotic adjustment in drought-stressed grapevines [35,36,37]. The present study aimed at investigating the impact of a short and severe drought stress on petiole anatomical traits and NSC content of SY and CS grapevines, in order to outline the cultivar-specific drought response strategies to cope with drought at petiole level.

## 2. Results

### 2.1. Water Status of Cabernet Sauvignon and Syrah Subjected to WATER DEFICIT

**Figure 1:**
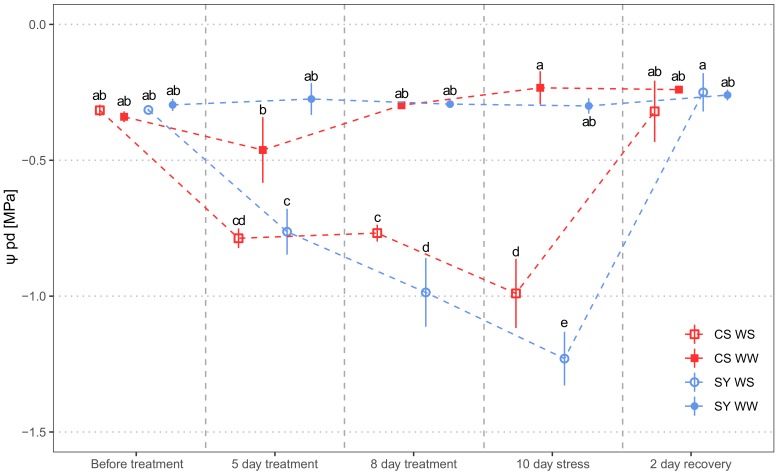
Predawn leaf water potential (Ψ_pd_) measured in Cabernet Sauvignon (CS) and Syrah (SY) vines under well-watered (WW) and water stress (WS) conditions. Measurements were carried out the day before water stress imposition, during stress and 2 days after rehydration, respectively. Different letters correspond to statistically significant differences (*p* <0.05).

Predawn leaf water potential (Ψ_pd_) was chosen as a representative of grapevine water status, with all other functional parameters (Section 4.1) being highly correlated (Figure S1). Water stress (WS) significantly affected the predawn leaf water potential (Ψ_pd_). No difference emerged between SY and CS, but the interaction treatment x cultivar evidenced contrasting responses of the cultivars to WS (Table 1). Ψ_pd_ in well-watered (WW) vines was similar in both cultivars over the whole experiment (Figure 1). As expected, drought induced a time-dependent decrease in Ψ_pd_. In particular, CS underwent a mild reduction in water status, since at Day 5 and 8 the Ψ_pd_ was similar, whereas SY suffered a larger and significant drop in Ψ_pd_. At the end of the water stress period (10th day), SY had more negative Ψ_pd_ than CS. Re-irrigation allowed a prompt recovery of Ψ_pd_ in both cultivars.

### 2.2. Petiole Xylem Anatomy and Theoretical Hydraulic Conductivity

**Figure 2:**
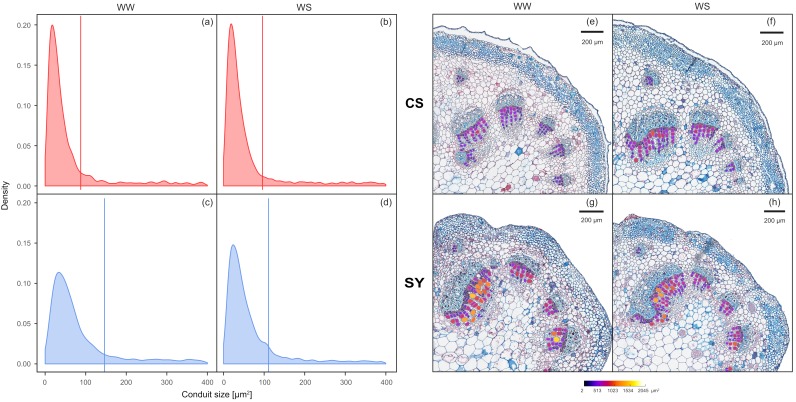
Left panel: frequency of xylem conduit size in petioles of Cabernet Sauvignon (CS) (**a**,**b**) and Syrah (SY) (**c**,**d**) vines under well-watered (WW) and water stress (WS) conditions. Lines correspond to the mean values of each distribution curve. Right panel: representative cross-sections of Cabernet Sauvignon (CS) (**e**,**f**) and Syrah (SY) (**g**,**h**) petioles, in well-watered (WW) and water stress (WS) conditions. The color scale indicates different conduit sizes (from dark blue for the narrower to white for the wider).

At the end of the experiment, the difference in the distribution of xylem conduit dimensional classes between CS and SY in WW vines was statistically significant, showing that SY had larger conduits than CS (Figure 2a,c,e,g; D = 0.14; *p* <0.001). Nonetheless, in WS vines, the mean size of conduits was similar in both cultivars (90 and 100 µm^2^ for CS and SY, respectively—Figure 2b,d,f,h; D = 0.29, *p* = *p* <0.001). In detail, the CS conduit size distribution was similar in WW and WS (Figure 2a,b,e,f; D = 0.02, *p* = 0.43). The distribution of conduit size in SY was significantly affected by the treatment (Figure 2c,d,g,h; D = 0.14, *p* <0.001), as WW vines had larger conduits, with higher mean size when compared to WS vines (~140 vs. 100 µm^2^, respectively).

Consistently with the above-described differences in conduit size, the theoretical specific hydraulic conductivity (K_ts_) also displayed a significant difference in the interaction time x cultivar, but did not reveal significant differences due to cultivar or time (Table 2). However, whereas in CS the K_ts_ was similar in all treatments, in SY it increased from 0.20 ± 0.08 Kg MPa^−1^ s^−1^ m^−1^ at time zero to 0.41 ± 0.22 Kg MPa^−1^ s^−1^ m^−1^ and 0.35 ± 0.04 Kg MPa^−1^ s^−1^ m^−1^ in WW and WS, respectively, at the end of the experiment.

### 2.3. Non-Structural Carbohydrates in Petioles

**Figure 3:**
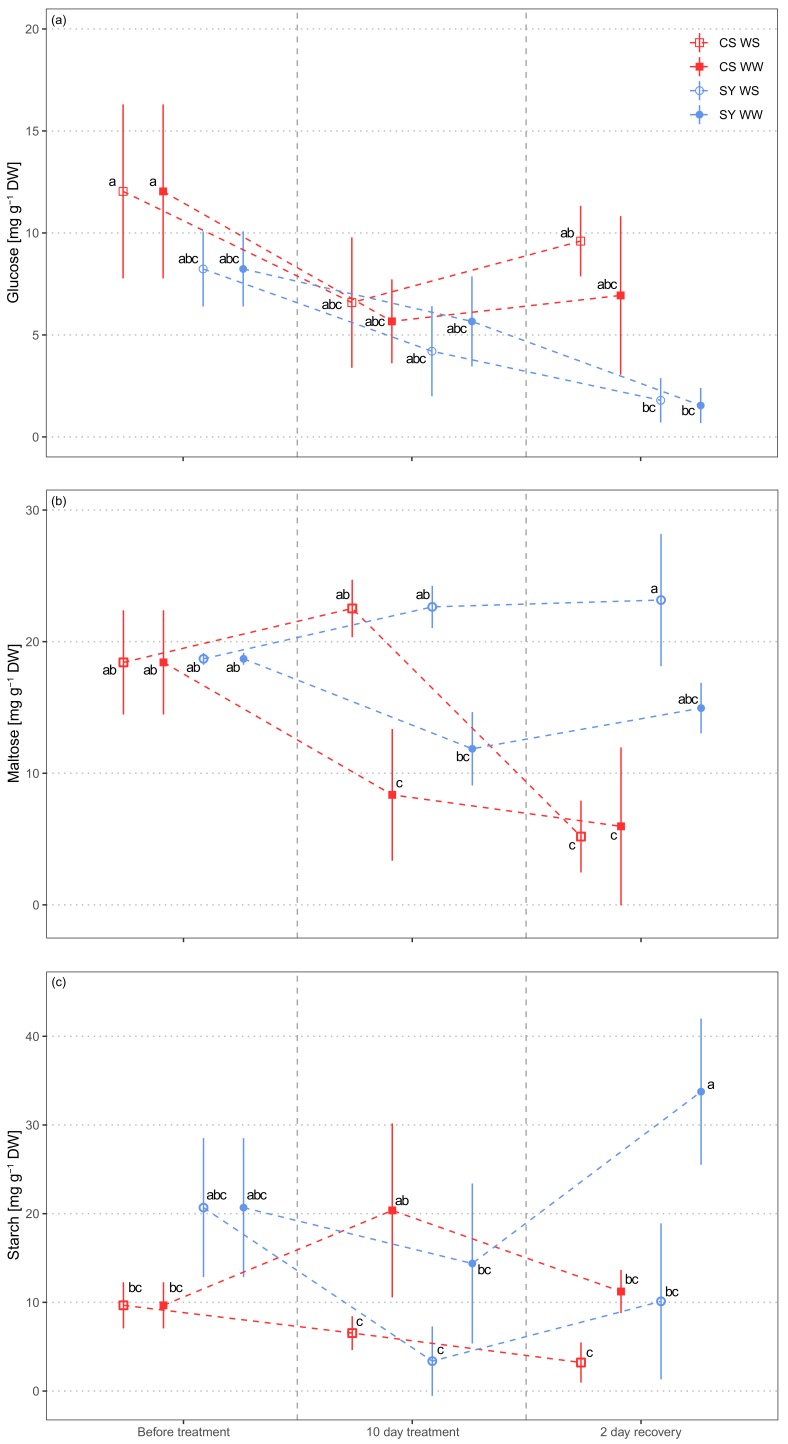
Non-structural carbohydrate (panel (**a**), glucose; panel (**b**) maltose; panel (**c**), starch) concentration in leaf petioles of Cabernet Sauvignon (CS) and Syrah (SY) vines under well-watered (WW) and water stress (WS) conditions. Different letters correspond to statistically significant differences (*p* <0.05).

The glucose concentration in petioles was different between the cultivars (Table 3). The concentration was higher in CS as compared to SY, and it significantly decreased during the experiment in both cultivars, especially in SY (Figure 3a).

Since sucrose and maltose concentrations were highly correlated (Figure S2), maltose was also used to represent sucrose. Maltose significantly changed in response to all variables and in several interactions (i.e., time × treatment, time × cultivar and treatment × time × cultivar) (Table 3). A decreasing trend in WW samples of both CS and SY was detected, with overall lower concentration in CS (Figure 3b). On the contrary, WS treatment induced an increase in maltose concentration in both cultivars compared to the control, with a larger increase in CS. However, after re-watering (2 days of recovery) the two cultivars showed opposite behavior, as maltose concentration returned to the same level as the controls in CS, while it did not change in SY.

The starch content also underwent significant changes in response to both treatment and cultivar (Table 3). In WS vines, starch concentration was significantly lower than in the control, regardless of the cultivar (Figure 3c), and even after 2 days from re-watering.

### 2.4. Relationships between Petiole NSC Content and Anatomy

**Figure 4:**
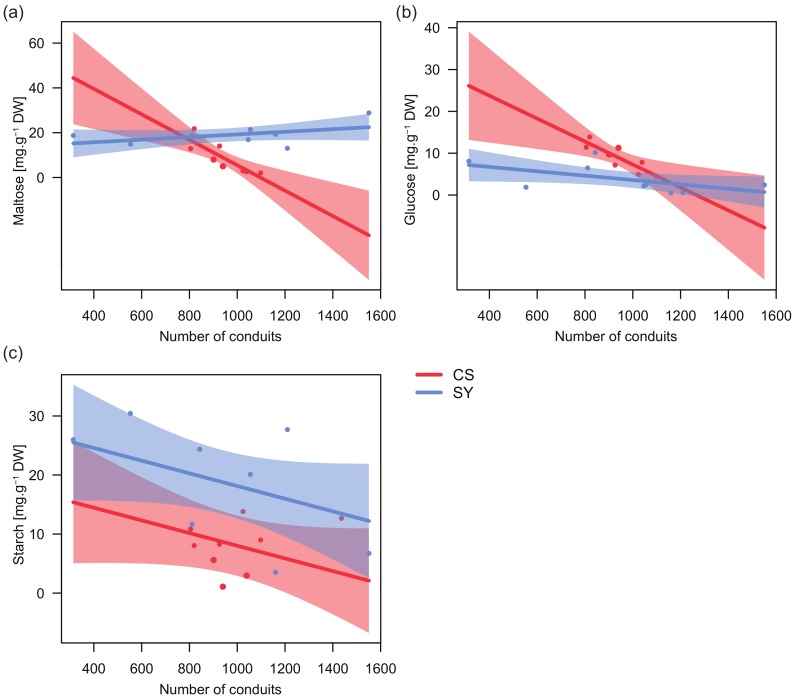
Relationships between the content of glucose (**a**), maltose (**b**) and starch (**c**) in leaf petiole and the number of conduits in Cabernet Sauvignon (CS) and Syrah (SY), according to the outcomes of applied linear models. Shaded areas represent the confidence intervals (0.95).

Significant relationships emerged between the concentration of both glucose and maltose, and the total number of petiole xylem conduits, with cultivar-specific trends (Table 4). In CS, the number of conduits was negatively correlated with the concentration of both glucose and maltose (Figure 4a,b), whereas in SY maltose and glucose were nearly unrelated to this anatomical trait.

On the contrary, in both cultivars the relationship between starch and the number of conduits showed similar trends, with a decreasing number of conduits along with increasing starch levels and starch concentration in SY always being higher than in CS (Figure 4c). Consistently, for starch, no significant interaction between the number of conduits and cultivar was found (Table 4).

### 2.5. Relationships between Petiole NSC and Ψ_pd_

**Figure 5:**
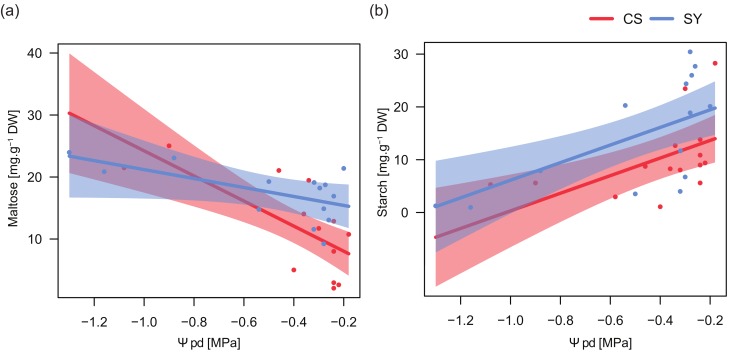
Relationships between predawn leaf water potential (Ψ_pd_) and maltose (**a**) and starch (**b**) content in leaf petioles of Cabernet Sauvignon (CS) and Syrah (SY), according to the outcomes of applied linear models (see text for details). Shaded areas represent the confidence intervals (0.95).

In the two cultivars, maltose content was related to changes in Ψ_pd_ with similar trends but different magnitudes (Table 5). Maltose concentration increased in petioles with low Ψ_pd_, especially in CS (Figure 5a). On the contrary, petiole starch content strongly decreased at low Ψ_pd_ in both cultivars.

## 3. Discussion

### 3.1. Water Status and Petiole Anatomy

Grapevine cultivars respond to water limitation by adopting specific hydraulic strategies, ranging from conservative to dissipative water use [26]. These contrasting adaptations involve various degrees of stomatal closure, xylem anatomical traits (size and number of xylem conduits), hormonal signaling, and eventual capacity for embolism recovery [38].

In grapevine, the leaves and petioles are the sectors most vulnerable to xylem embolism, acting as safety valves to protect xylem integrity in other organs, like the roots and stem [3,39,40]. As a result, hydraulic segmentation might lead to daily or seasonal cycles of embolism events during water shortage periods in leaves and petioles, and the subsequent recovery of hydraulic functioning. Regardless of the mechanisms proposed [40], xylem refilling is an energy-demanding process and it is supposed to require starch reserve mobilization [24,41]. In the present study, we compared petiole anatomical traits and NSC content of two grapevine cultivars (CS and SY) subjected to either water stress (WS) or well-watered (WW) regimes.

The results confirm that a ten-day-long water limitation imposed to CS and SY potted grapevines strongly influenced leaf gas exchanges (result not shown) and water status, as shown by the drop in Ψ_pd_. Although the isohydric/anisohydric classification should be cautiously assumed [42], a more marked Ψ_pd_ drop under drought would be expected in SY, since it is supposed to be a near-anisohydric cultivar [43,44,45]. In contrast, CS is generally recognized as isohydric [33,43,46,47] and hence should show higher Ψ_pd_ during stress treatment. Consistently, our analysis revealed some varietal differences, since SY exhibited a more negative Ψ_pd_ compared to CS, supporting the hypothesis that SY is less prone to rapid stomatal closure under water shortage.

According to the literature [48,49], a close relationship between xylem architecture and response to water stress has been proposed, although other factors could also play significant roles. In grapevine, a strong correlation was demonstrated between stem diameter, xylem conduit diameter and hydraulic properties by comparing young shoots and mature trunks [50]. In the case of petioles, a high proportion of small conduits is expected to lead to lower theoretical hydraulic conductivity. However, at time zero, there was no significant difference in K_ts_ between CS and SY, since the small size of conduits in CS was counterbalanced by their higher number, as compared to SY (see also Figure S2, being average conduit area inversely related to conduit number). On the other hand, SY petioles showed wider conduits, as compared to CS. In response to drought, SY showed a significant reduction in the frequency of large xylem conduits, which could be ascribed to a shift in the petiole cambium differentiation. As a result, new conduits formed with a size similar to that observed in CS. These observations would support the hypothesis that SY could be more vulnerable to embolism when exposed to water limitation [51], whereas CS could be anatomically pre-adapted to water stress, exhibiting constitutively smaller conduits. These findings are consistent with reports by Netzer et al. [49] and Munitz et al. [48], which described cambial activity, conduit diameter and hydraulic conductance in response to limited water availability. On the other hand, SY showed increased K_ts_ in both WW and WS at the end of the experiment. Nevertheless, this parameter was not different between cultivars, because the drought-driven production of smaller conduits in SY was compensated by their increased number. Our results suggest the existence of a cultivar-specific plasticity of cambial cells, leading to the production of different dimensional classes of xylem conduit. This also implies that a well-coordinated developmental process in SY is likely to contribute to the maintenance of the water balance when drought episodes occur during the season. Taken together, these analyses confirm not only that the petiole xylem anatomy in SY is different from that of CS, but also that the varietal responses to hydraulic impairment could be ascribed to a change in cambial differentiation at the bundle level, ultimately affecting conduits dimension and distribution.

### 3.2. Petiole Non-Structural Carbohydrates

The controversial role of NSC pools in the plant response to drought, due to the interaction and balance of these pools between plant organs and tissue partitioning at different time scales [52,53], prompted us to compare petiole NSC content in water stressed and well-watered grapevines, in order to elucidate their possible changes under drought and recovery.

Our results confirm significant differences in NSC concentration in the petioles of the two cultivars. Starch storage represents the overall metabolic response, but it is apparently not directly related to glucose [54]. Correspondingly, we show that glucose and starch pools are not related and, since glucose did not even correlate with maltose/sucrose (Figure S2), we could argue that its concentration mirrors the signaling [55,56] and energetic status [57] requirements. Therefore, the decrease in glucose observed during the experiment could indicate a reduction in metabolic activity in the tissues.

The relationship between starch reserves and maltose content is crucial to explaining the physiological drought response based on the osmotic control of the decreasing water potential, as already investigated by Patakas et al. [36]. In our experiment, the drop in leaf water potential in stressed plants was counteracted by an increase in maltose/sucrose in both cultivars, which may modify the osmotic potential, thus allowing turgor maintenance. Interestingly, CS-stressed plants exhibited an almost complete depletion of maltose at the recovery stage, whereas SY displayed steady levels of the same sugar. This observation supports the hypothesis that CS stress response could be mainly based on NSC utilization, whereas, in SY, anatomical adjustments would also account for drought tolerance. On the other hand, in both SY and CS stressed plants, the levels of starch did not show any significant change at the recovery stage. In fact, the modifications induced by drought could not be reversed in a short time by the restoration of the water supply, similarly to what was observed in Mediterranean trees recovering from severe drought stress [58].

### 3.3. NSC Content, Water Status and Xylem Architecture as an Integrated Cultivar-Specific Response to Drought

The relationships between NSC content and predawn leaf water potential (Table 5 and Figure 5) supported the putative role of NSC dynamics in plant responses to drought. Both cultivars displayed a similar trend in petiole starch concentration in response to Ψ_pd_ variations. Conversely, maltose/sucrose accumulation showed a significant difference between cultivars (Table 5) and the concentration of these sugars, being related to the magnitude of stress, displayed in CS a much more pronounced negative relationship. On the other hand, SY exhibited an intermediate behavior between anisohydric and isohydric extremes, possibly taking advantage of the osmotic effect of maltose/sucrose to face drought stress, although this was not the only response strategy adopted by this cultivar. It could suggest the involvement of maltose in the recovery process; this hypothesis is supported by the significantly higher content of maltose in SY during the recovery stage.

Starch reserves and the number of conduits were negatively correlated in both cultivars (Figure 4). This can be explained by the inverse correlation, found in petioles, between xylem conduit number and the area occupied by parenchyma (not shown, r = −0.61; *p* <0.05). In fact, a solid relationship between total parenchyma and stem starch reserves has been demonstrated in trees [59]. This feature could indicate that a higher carbon investment in xylem conduits determines a lower capacity for the storage of reserves.

Variations in xylem architecture have been discussed in relation to hydraulic behavior in response to water shortage [60]. Grapevine cultivars showing wider xylem conduits are believed to have higher hydraulic conductance and xylem vulnerability [43]. In CS, low levels of glucose and maltose/sucrose corresponded to a higher number of conduits (Figure 4) and consequently to a higher translocation area (Figure S2). Conversely, in SY, where the increase in conduit number was counterbalanced by the decrease in their diameter and starch reserves were not limiting, it is likely that both metabolic substrates and osmolites remained almost unchanged.

## 4. Materials and Methods

### 4.1. Plant Material and Experimental Design

The experiment was carried out at the experimental farm of the University of Udine, located in the Friuli Venezia Giulia region (North-Eastern Italy; 46°02′ N, 13°13′ E; 88 m a.s.l.). Twenty-four vines of cv. Cabernet Sauvignon R5 (*Vitis vinifera* L.) and cv. Syrah ISV-R1 (*Vitis vinifera* L.), two years old, both grafted on S.O.4, were planted in March 2016 in 20 L plastic pots, filled with field sieved soil supplemented with 10% perlite. The plants were grown under a clear plastic film tunnel (EVA ethylene-vinyl-acetate) opened on the sides as described in Herrera et al. [61]. The plants were organized in a completely randomized experimental design encompassing three rows. During the growing season, the vines were trained vertically and when the shoot length exceeded the height of the trellising system (2.1 m), the shoot was positioned horizontally in the last catching wire. Water was supplied by a drip irrigation system with three emitters per pot (PCJ 2 L h^−1^, Netafim, Israel) and a set of vines was positioned under weighting mini-lysimeters to measure evapotranspiration (ETc), as described in Hochberg et al. [27]. During the season, the irrigation was maintained at 100% ETc, until treatments were imposed from 8–18 August: WW, well-watered vines; WS, water-stressed vines (irrigation withheld by removing the drippers from the pots). At the end of the stress treatment, the water-stressed vines were re-irrigated at field capacity and samples were harvested two days later.

The predawn leaf water potential (Ψ_pd_) and stem water potential (Ψ_stem_) were measured (at 5 time-points) on fully expanded leaves at 6 am and 1 pm, respectively. To measure the water potential, the leaves were bagged immediately (for Ψ_pd_) or 1 h for (Ψ_stem_) before measurement and they were excised from the shoot using a sharp blade. The bagged leaves were placed into the pressure chamber (Soil Moisture Co., Santa Barbara, CA, USA), pressurized using a nitrogen tank, and Ψ was recorded when the initial xylem sap was observed emerging from the cut end of the petiole. The LI-6400 portable photosynthesis system (Li-Cor Inc., Lincoln, NE, USA) device was used to measure photosynthesis (P_N_; mmol CO_2_ m^−2^ s^−1^), stomatal conductance (*g*_s_; mol H_2_O m^−2^ s^−1^) and transpiration (E; mmol H_2_O m^−2^ s^−1^) using a constant light intensity (1000 μmol photons m^−2^ s^−1^) and CO_2_ concentration (400 μmol mol^−1^). The leaf water potential and gas exchange measurements (three replicates x treatment) were carried out on clear and sunny days at the beginning of the trial before the imposition of stress, during water stress and after recovery on both SY and CS vines and both WW and WS treatments. K_LEAF_ was calculated as E/(Ψ_pd_ − Ψ_stem_)_._

### 4.2. Anatomical Measurements and Theoretical Hydraulic Conductivity

Petioles from leaves sampled for Ψ_pd_ measurement were separated into two portions; the basal portion was used for that quantification of non-structural carbohydrates (NSCs), and the distal portion was used for anatomical analysis. Sampling was performed before dawn, and therefore the glucose and maltose/sucrose levels in petioles were not dependent on photosynthetic activity. Consistently, after a whole night, the starch level was also at a minimum [62], being the sum of residual transitory starch and of secondary starch. After sampling, the cross-sections of petioles were processed as described by Falchi et al. [34]. Briefly, 6-µm microtome sections (stained with safranin/alcyan blue) were prepared (see Figure 2 as example) and three digital slides for each cultivar and for each treatment were acquired with an Aperio CS scanner (Leica, model AT2, Wetzlar, Germany) at 5X magnification and anatomical parameters were analyzed by open-source Fiji software [63]. The following parameters were analyzed: conduit number, total conduit area, average conduit area.

The theoretical hydraulic conductivity (K_t_; Kg MPa^−1^ s^−1^) was estimated using the Hagen–Poiseuille’s modified by Tyree and Ewers (1991) as ρπ 128 η^−1^ Σ D^4^, where D is the radius of the conduit in meters, ρ is the fluid density (assumed to be 1000 Kg m^−3^) and η is the viscosity (assumed to be 8.9 × 10^−10^ MPa). The theoretical specific hydraulic conductivity (K_ts_; Kg MPa^−1^ s^−1^ m^−1^) was calculated by dividing K_t_ by the cross-sectional area of the petiole (PA).

### 4.3. Non-Structural Carbohydrates (NSC)

The extraction and analysis of carbohydrates followed the protocol of Quentin et al. [64] adapted to small amounts of material, as described by Savi et al. [65]. Petioles were immediately microwaved for 2 min at 600 W to block enzymatic activities. To avoid the loss of NSC due to tissue burning, the operation was made in a water-sutured space. Further samples were dried at 60 °C overnight. Then, they were ground into fine powder in a mortar under liquid nitrogen. Aliquots of 20 ± 2 mg of powder were suspended in 0.3 mL ethanol 80% (*v*/*v*) twice, incubated at 80 °C for 30 min, and then dried at 60 °C overnight. A third suspension was made with 0.5 mL of 50 mM Tris-HCl pH 7.5 and used to re-suspend the crystalized carbohydrates resulting from previous steps. The supernatants obtained after incubation at 80 °C and a 14,000× *g* centrifugation for 5 min were used for soluble sugar measurement, while the pellet was re-suspended in 1 mL of acetate buffer (0.4 M NaCH_3_COO, pH 4.6), and used for starch quantification. Glucose was quantified in a VICTOR3 Multilabel Counter Plate Reader (Perkin Elmer, Boston, MA, USA) after enzymatic conversion to NADPH by 0.1 U per sample of hexokinase and glucose−6-P dehydrogenase at 37 °C for 10 min in 100 µL of assay buffer (50 mM Tris-HCl pH 7.5 with 5 mM MgCl_2_ and 125 µM NADP^+^). For sucrose analysis, 100 µL of the supernatant was placed in a 1.5 mL Eppendorf tube with 300 µL of acetate buffer with 25 U of invertase to break down sucrose into fructose and glucose. The same procedure was followed for soluble starch degradation products—maltose disaccharide fraction and maltodextrin oligosaccharides—using 25 U of α-amyloglucosidase per sample (in the text maltose was used to indicate this pool of sugars coming from starch degradation). The amount of soluble carbohydrates was then expressed as mg glucose g^−1^ DW by using a calibration curve of known amounts of glucose.

The starch contained in the pellets was digested overnight at 55 °C with 100 U α-amylase and 25 U α-amyloglucosidase per sample. The amount of starch (mg g^−1^ DW) was calculated by means of a calibration curve made with the known amounts of amylose from potato starch (all the chemicals was form Sigma-Aldrich, Milan, Italy).

### 4.4. Statistical Analysis

The effect of treatment on the functional parameters of the two cultivars was tested using a three-way analysis of variance (ANOVA). As all the parameters related to plant water status were highly correlated (r > 0.7; *p* <0.001; Figure S1), we used the predawn leaf water potential (Ψ_pd_) as the dependent variable and the treatment, cultivar, time and their interactions as fixed factors.

The distribution of xylem conduit size was compared between grapevine cultivars, before and after the treatment, and within each cultivar, for water-stressed vs. well-watered plants. The xylem conduit distribution was represented by density plots. Paired differences in conduit size distribution were tested by the two-sample Kolmogorov-Smirnov test (*p* <0.05).

Variations in K_ts_ between cultivar, treatment, time after treatment and their interactions were tested using a three-way analysis of variance (ANOVA).

Preliminarily, the correlations of measured NSC (i.e., glucose, maltose, sucrose and starch) were assessed by Pearson test. Due to the high collinearity between sucrose and maltose (r > 0.7, *p* <0.001) and sucrose and starch (r > 0.4, *p* <0.001) (Figure S2), sucrose was considered as interchangeable with maltose content and was not included in the box plot of NSC concentration (Figure 2). The variations in NSC between cultivar, treatment, time after treatment and their interactions were tested using a three-way analysis of variance (ANOVA).

The relationships between NSC content (i.e., glucose, maltose and starch) and petiole xylem anatomy in the two cultivars were tested using linear models. The conduit number was used as a proxy of anatomy variation, as it showed high correlation with the other parameters (Figure S2). Each model included NSC as the dependent variable and grape cultivar, number of conduits and their interaction as fixed effects. Consistently, the content of NSC was also related to predawn leaf water potential, comparing the interaction with the two cultivars. The models were simplified with a manual backward stepwise procedure, where significance was missing (*p* > 0.05).

All the statistical analyses were performed in R statistical software [66] and model assumptions (i.e., linear models and ANOVA) were checked by diagnostic plot of residuals. Outlier records were detected by the function “outlier Test” of the ‘car’ package [67] and removed from models (where *p* <0.01).

## 5. Conclusions

According to most of the traits analyzed in the present study, we suggest that CS and SY cultivars showed different strategies to face water shortage, even though a similar pattern of changes in predawn leaf potential was observed. CS was characterized by a lower content of starch in petioles and smaller xylem conduits when compared to SY, and its response to drought was mainly based on the accumulation of maltose/sucrose deriving from starch mobilization. In addition to NSC involvement in drought stress response and during recovery, SY also exhibited significant anatomical modifications, decreasing the size of conduits in the newly differentiated xylem. Since SY’s and CS’s strategy to face drought cannot be clearly classified, these results are in agreement with the recent criticism arisen on isohydric/anisohydric classification [8,9].

Furthermore, this study highlighted that the accumulation of maltose in petioles of water-stressed vines parallels the decrease in water potential, as already reported in poplar [24] and grapevine stems [34]. In particular, this behavior was more pronounced in CS under water stress and suggests a possible involvement of sugars in xylem refilling. This hypothesis needs further investigation aimed at clarifying the occurrence of embolism and the recovery from hydraulic failure in grapevine.

## Figures and Tables

**Table 1:** Outcomes of the analysis of variance (three-way ANOVA) applied to predawn leaf water potential (Ψ_pd_) in relation to drought stress (treatment), time, cultivar and their interactions.

Factor	Df	F Value	*p*-Value
Treatment	1,33	447.92	<0.001
Time	4,33	75.74	<0.001
Cultivar	1,33	0.80	0.386
Treatment × time	4,33	89.22	<0.001
Treatment × cultivar	1,33	9.27	0.004
Time × cultivar	4,33	7.36	<0.001
Treatment × time × cultivar	4,33	2.74	0.045

**Table 2:** Theoretical specific hydraulic conductivity (K_ts_) in relation to drought stress (treatment), time, cultivar and their interactions. Data were analyzed with a three-way ANOVA.

Factor	Df	F Value	*p*-Value
Treatment	1,16	0.004	0.952
Time	1,16	0.998	0.332
Cultivar	1,16	0.134	0.719
Treatment × time	1,16	0.004	0.952
Treatment × cultivar	1,16	0.467	0.504
Time × cultivar	1,16	64.909	0.021
Treatment × time × cultivar	1,16	0.467	0.504

**Table 3:** Analysis of variance (three-way ANOVA) applied to leaf petiole non-structural carbohydrates content in relation to drought stress (treatment), time, cultivar and their interactions. Statistically significant values are marked in bold.

Factor	Df	F Value	*p*-Value
*Glucose*			
Treatment	1,24	0.193	0.664
Time	2,24	13.239	<0.001
Cultivar	1,24	18.454	<0.001
Treatment × time	2,24	0.355	0.705
Treatment × cultivar	1,24	0.787	0.384
Time × cultivar	2,24	3.007	0.068
Treatment × time × cultivar	2,24	0.197	0.823
*Maltose*			
Treatment	1,22	23.922	<0.001
Time	2,22	13.237	<0.001
Cultivar	1,22	16.455	<0.001
Treatment × time	2,22	8.667	0.002
Treatment × cultivar	1,22	0.823	0.374
Time × cultivar	2,22	13.442	<0.001
Treatment × time × cultivar	2,22	3.504	0.048
*Starch*			
Treatment	1,2	24.259	<0.001
Time	2,22	1.287	0.296
Cultivar	1,22	11.684	0.002
Treatment × time	2,22	6.383	0.006
Treatment × cultivar	1,22	1.029	0.321
Time × cultivar	2,22	10.935	<0.001
Treatment × time × cultivar	2,22	3.651	0.043

**Table 4:** Outcomes of the linear models (ANOVA table) applied to non-structural carbohydrates (i.e., glucose, maltose, starch) in relation to xylem conduit number, cultivar and their interaction.

Explanatory variables	Df	F Value	*p*-Value
*Glucose*			
Conduit number	1,13	7.91	0.015
Cultivar	1,13	15.38	0.002
Conduit number × cultivar	1,13	5.21	0.040
*Maltose*			
Conduit number	1,13	0.25	−
Cultivar	1,13	26.01	0.000
Conduit number × cultivar	1,13	16.23	0.001
*Starch*			
Conduit number	1,14	4.33	−
Cultivar	1,14	8.39	0.012
Conduit number × cultivar	−	−	−

**Table 5:** Outcomes of the linear models (ANOVA) applied to non-structural carbohydrates in relation to predawn leaf water potential (leaf potential), cultivar and their interaction.

Explanatory variables	Df	F Value	*p*-Value
*Glucose*			
Leaf potential	−	−	−
Cultivar	1,26	7.13	0.013
Leaf potential × cultivar	−	−	−
*Maltose*			
Leaf potential	1,23	19.21	0.000
Cultivar	1,23	5.59	0.027
Leaf potential × cultivar	1,23	4.45	0.046
*Starch*			
Leaf potential	1,26	11.21	0.002
Cultivar	1,26	5.59	0.049
Leaf potential × cultivar	−	−	−

## Supplementary Materials

Supplementary materials can be found at https://www.mdpi.com/1422-0067/21/4/1457/s1.
