# Supplementary material for: Analysis of Non-Structural Carbohydrates and Xylem Anatomy of Leaf Petioles Offers New Insights in the Drought Response of Two Grapevine Cultivars
Source: Int J Mol Sci. 2020 Feb 20;21(4):1457. doi: 10.3390/ijms21041457 (PMC7073087; doi:10.3390/ijms21041457)
Supplement: Supplementary file 1 [file ijms-21-01457-s001.pdf]

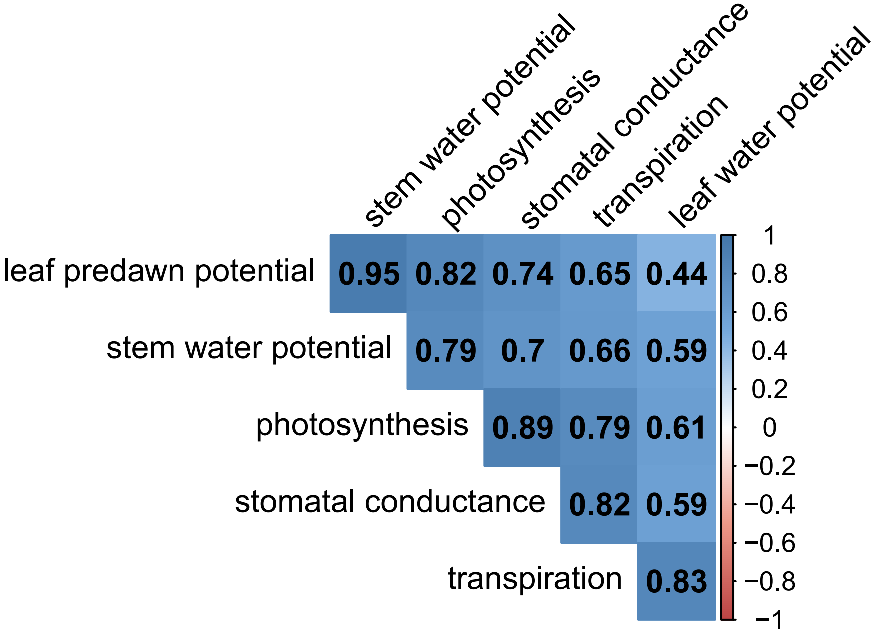

Figure S1. Correlations between the measured hydraulic parameters. Numbers represent Pearson's r (p<0.001)

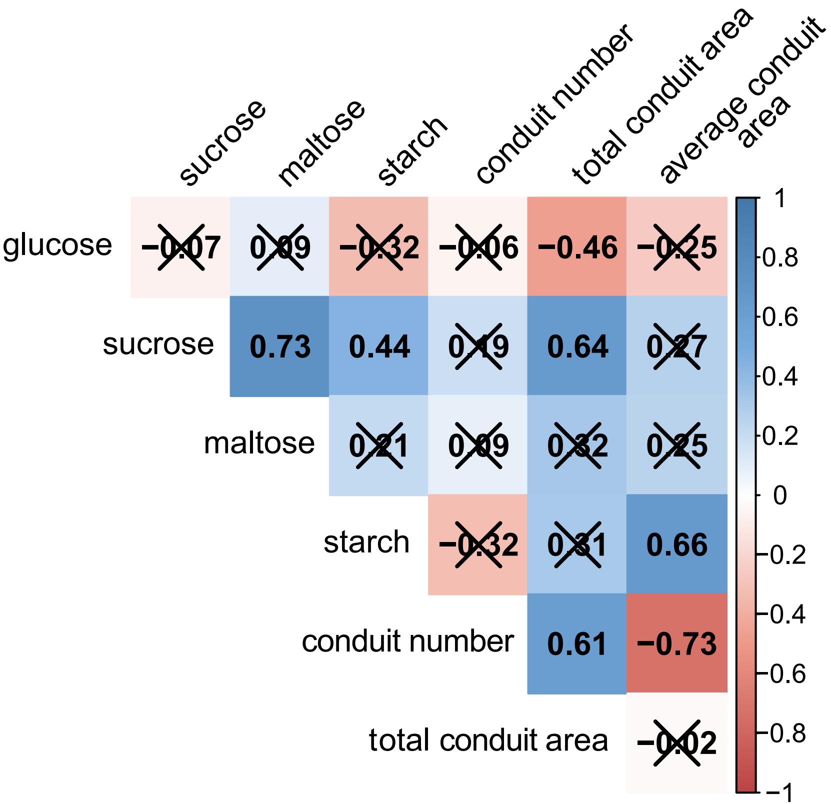

Figure S2. Correlations between the concentration of non-structural carbohydrates and xylem conduit measurements. In blue, positive correlations; red, negative correlations. Numbers represent Pearson's r, not significant correlations are also marked with a cross symbol ($p<0.05$)
